# Resource allocation in NHS dentistry: recognition of societal preferences (RAINDROP): study protocol

**DOI:** 10.1186/s12913-018-3302-8

**Published:** 2018-06-22

**Authors:** Christopher R. Vernazza, Katherine Carr, John Wildman, Joanne Gray, Richard D. Holmes, Catherine Exley, Robert A. Smith, Cam Donaldson

**Affiliations:** 1 Centre for Oral Health Research, Newcastle University, Framlington Place, Newcastle upon Tyne, NE2 4BW UK; 2 Newcastle University Business School, Newcastle University, Room 7.20, 7th Floor, 5 Barrack Road, Newcastle upon Tyne, NE1 4SE UK; 3 Nursing, Midwifery & Health, Northumbria University, Sutherland Building, Newcastle-upon-Tyne, NE1 8ST UK; 4 Faculty of Health and Life Sciences, Northumbria University, NB266, 2nd Floor, Northumberland Building, Newcastle upon Tyne, NE1 8ST UK; 5 ScHARR, University of Sheffield, Regent Court, 30 Regent Street, Sheffield, S1 4DA UK; 6 Yunus Centre for Social Business and Health, Glasgow Caledonian University, M201, George Moore Building, Glasgow, G4 0BA UK

**Keywords:** Health economics, Priority setting, Preference elicitation, Oral health

## Abstract

**Background:**

Resources in any healthcare systems are scarce relative to need and therefore choices need to be made which often involve difficult decisions about the best allocation of these resources. One pragmatic and robust tool to aid resource allocation is Programme Budgeting and Marginal Analysis (PBMA), but there is mixed evidence on its uptake and effectiveness. Furthermore, there is also no evidence on the incorporation of the preferences of a large and representative sample of the general public into such a process. The study therefore aims to undertake, evaluate and refine a PBMA process within the exemplar of NHS dentistry in England whilst also using an established methodology (Willingness to Pay (WTP)) to systematically gather views from a representative sample of the public.

**Methods:**

Stakeholders including service buyers (commissioners), dentists, dental public health representatives and patient representatives will be recruited to participate in a PBMA process involving defining current spend, agreeing criteria to judge services/interventions, defining areas for investment and disinvestment, rating these areas against the criteria and making final recommendations. The process will be refined based on participatory action research principles and evaluated through semi-structured interviews, focus groups and observation of the process by the research team. In parallel a representative sample of English adults will be recruited to complete a series of four surveys including WTP valuations of programmes being considered by the PBMA panel. In addition a methodological experiment comparing two ways of eliciting WTP will be undertaken.

**Discussion:**

The project will allow the PBMA process and particularly the use of WTP within it to be investigated and developed. There will be challenges around engagement with the task by the panel undertaking it and with the outputs by stakeholders but careful relationship building will help to mitigate this. The large volume of data will be managed through careful segmenting of the analysis and the use of the well-established Framework approach to qualitative data analysis. WTP has various potential biases but the elicitation will be carefully designed to minimise these and some methodological investigation will take place.

**Electronic supplementary material:**

The online version of this article (10.1186/s12913-018-3302-8) contains supplementary material, which is available to authorized users.

## Background

In any healthcare system, the problem of scarcity exists, in that there are insufficient resources (such as monetary funds, staff time, estates space) to deliver all of the possible programmes and interventions [1]. Where resources are used to provide one service or intervention, there is an opportunity costs in terms of the benefit forgone by using these resources for an alternative service [2]. There is therefore a need to allocate resources in a way that meets the objectives of the health system, however these are defined [3]. This is often difficult as healthcare systems usually have complex and sometimes competing objectives.

Different healthcare systems have dealt with this need to undertake resource allocation in different ways, either explicitly or implicitly, with varying degrees of success in terms of maximising benefits. Methods of resource allocation are varied but do not usually address the opportunity costs associated with the decisions in a systematic way [3]. Examples of these methods include perpetuating historical allocations to different service areas and diverting funds to those areas where there is political, patient or clinician pressure (with those able to make their voice heard better placed to secure funds). A final example which also does not address opportunity cost would be allocating funds based on assessment of needs or defining core services. Methods developed in the field of health economics, such as cost-effectiveness and cost-utility analysis, have been used, but these address the performance of each intervention relative to cost and often do not actually address resource allocation issues. This usually results in multiple interventions being recommended with no consideration of the overall budget (i.e. where the resource will come from) or with only several alternatives being compared rather than holistically looking across a whole system [4]. A more pragmatic approach has been suggested which involves economics-based priority setting tools, an example of which is Programme Budgeting Marginal Analysis (PBMA) [5]. In this approach, the current spend in different areas is defined, areas for investment and disinvestment are identified and these are judged against criteria that are developed as part of the process to encapsulate benefit before recommendations on where to disinvest and where to invest are made.

In a review of PBMA applications between 1991 and 2009, although 65% had led to some change in actual resource allocation, ongoing use of PBMA after the academic study was achieved only in 22% of cases [6]. A further review of all explicit priority setting techniques between 2000 and 2017 echoed the lack of ongoing use and ascribed this to difficulties in the complexity and time burden of using the various frameworks [7].

Although the involvement of the public and the incorporation of their views in priority setting processes has been recognised as vital, typically only unrepresentative small groups of the public or single individuals have been involved in decisions. There have been only limited attempts in health to systematically elicit public preferences for use in commissioning decisions [8]. One particular economic tool, willingness to pay (WTP) could be used to systematically elicit of the views of a large sample of the public. Although incorporating systematically elicited WTP values from large samples into resource allocation processes has been postulated as best practice [9], there is no evidence that this has been done in any country or setting.

The need for priority setting tools is therefore clear and PBMA is a well-accepted tool, but there are challenges particularly with ongoing use and the input of the public. The study therefore aims to undertake a large scale PBMA whilst studying and addressing these concerns.

The chosen exemplar area for the study is National Health Service (NHS) dentistry in England. The spend on this is around £3.7 billion per year or 3.5% of the NHS budget [10]. Currently resource allocation is mostly based on historical allocations to individual dental practices or dentists. In addition, the standard contract with dental practices which stipulates what dentists should use this resource on is inflexible and not sensitive to demands from commissioners [11]. The case for more effective resource allocation was made strongly in an independent review of NHS dentistry [12].

Major changes in the commissioning of NHS care in England introduced as part of the Health and Social Care bill in 2009 took effect in April 2013. Essentially for dentistry, this meant that commissioning was removed from local bodies (Primary Care Trusts) to a national body (the National Commissioning Board, now referred to an NHS England) with part devolvement to local offices [10]. In order to be ready for the changes in April 2013, the board agreed to maintain a “steady state” initially, commissioning all services that had previously been commissioned. It has also committed to commissioning more intelligently as the new structure becomes more stable and there is some evidence that this is now beginning to happen [13]. As part of this more intelligent commissioning, the need to identify potential frameworks that commissioners can use to inform and make allocation decisions has been re- emphasised [10].

This research is therefore intended to apply PBMA to a national level programme (using dental services in England as the exemplar) incorporating the use of WTP values and to evaluate the process’s feasibility, utility and applicability from the perspective of the stakeholders involved.

## Methods/design

The aim of the study is to develop and use a framework which takes into account public values to inform resource allocation decisions to address the problem of scarce resources in health using NHS dentistry as an exemplar.

**Fig. 1:**
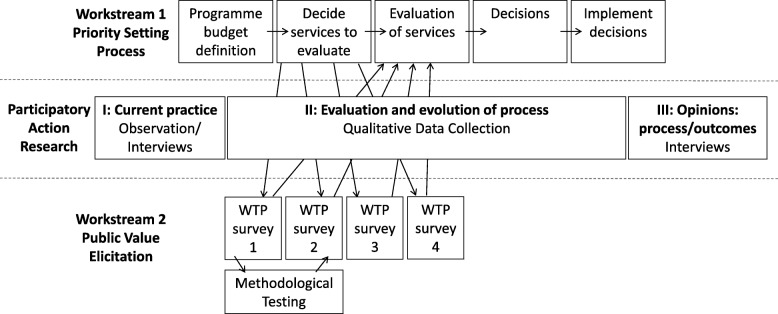
Overview of workstreams and Participatory Action Research

The study design is a mixed-methods study adopting a participatory action research (PAR) approach [14]. The design consists of two workstreams illustrated in Fig. 1; the first will involve participants undertaking a PBMA with the process continually evolving in response to the participants; the second will involve elicitation of societal preferences for different dental services being considered in WS1 using WTP from a representative sample of the public and then feeding the findings back into WS1.

### Workstream 1 (WS1)

This will take place in three phases:

I. pre-PBMA interviews with commissioners
II. application and monitoring of the PBMA process
III. review of PBMA process

The success and failures will be studied using the principles of PAR, which studies a community’s response to (or actions in relation to) a problem or situation and involves the participants as co-researchers, drawing on a variety of evidence to reflect on the process undertaken. This project will particularly employ the participative and reflective elements of PAR [15].

### Sample

For Phase I, the sample will be drawn from the NHS England commissioners responsible for leading dental commissioning in sub-regional areas. For Phase II, the panel will be drawn from NHS England dental commissioners, dental professionals (in the form of Local Dental Network (LDN) chairs, who advise on commissioning), Consultants in Dental Public Health (CDPH) and patient/public representatives. For Phase III, a sub-sample of the panel and a sub-sample of WS2 participants (who were involved in the WTP survey) will be drawn. For all 3 phases, sampling will be purposive with the aim to ensure representation of different roles, different regions and different genders. For interviews and focus groups in all phases, the principle of saturation will be used to determine sample size.

### Recruitment

For Phase I, all dental lead commissioners will be contacted by email and invited to participate. For Phase II/III, the study will be explained during a commissioning seminar to be delivered to all dental lead commissioners and LDN chairs and those attending will be invited to participate. To recruit CDPHs, those previously involved in commissioning and priority setting work will be identified and approached by email. For patient/public representatives, an existing patient advisory group will be approached by speaking at one of their meetings. The participants for the public group in Phase III will be recruited by asking those completing the questionnaires in WS2 if they wish to be contacted about WS1. Those indicating they are interested will be approached on a selective basis in order to fulfil purposive requirements.

### Intervention: Phase I

The first phase will correspond with the “plan” stage of PAR.

Selected members of the panel will take part in semi-structured interviews which will focus on current areas of concern about the commissioning process in NHS dentistry as well as facilitators and barriers to making and implementing resource allocation decisions.

### Intervention: Phase II

The panel will then meet, facilitated by the research team, to tailor the PBMA process. The exact nature of the exercise will depend on the needs of the panel but the general principles will be consistent with the seven steps of micro-PBMA as outlined below [16].

1. Determine the aim and scope of the priority setting exercise
2. Compile a “programme budget” The current resources used and activity information are identified.
3. Form a “marginal analysis” advisory panel
4. Determine relevant decision-making criteria and weighting these e.g. maximising benefits, improving access and equity, etc., with reference to specified objectives of the health system and the community.
5. Identify options for (a) service growth (b) resource release from gains in operational efficiency (c) resource release from scaling back or ceasing some services
6. Evaluate investments and disinvestments in terms of costs and the criteria established in Step 4 and make recommendations for (a) funding growth areas with new resources (b) moving resources from 5(b) and 5(c) to 5(a).
7. Validate results and reallocate resources

At the point of establishing options to be assessed in marginal analysis (Step 5), as well as continuing on through the ‘normal’ process of weighting criteria and assessing each option against these criteria, the options will be fed into WS2, in which each option will be assessed in terms of the mean societal value expressed as WTP. Once Step 6 is reached by the panel, the results of WS2 will be fed back into the process. WTP values for the programmes will be presented in a variety of ways and the panel will decide on what form of WTP presentation is most useful. The panel will also have to decide how much weight the WTP values will be given in any decisions made.

Phase II will correspond with the ‘Act & Observe’ phase of PAR which involve not only conducting the PBMA as outlined above but also collecting data and reflecting on the application of the framework, focusing on how the panel manage the process devised.

### Intervention: Phase III

Finally, the ‘Reflect’ phase of PAR corresponds to evaluation from participants relating their experiences, barriers, and successes. This evaluation will principally consist of another set of semi-structured interviews with panel members and two focus groups, one of the panel and one of members of the public (a sub-sample of those involved in WS2), which will focus on any differences in priorities. The results of this evaluation will be used to develop the framework further as a specific and original tool for dental decision-making, which can be improved further and adapted to other areas of health.

### Data collection and analysis

The data collected will be qualitative in nature and will consist of material gathered in interviews, focus groups and from field notes. The interviews and focus groups will be conducted using a topic guide which will evolve following each interview/focus group. The topic guide for Phase 1 has already been developed (Additional file 1) but further topic guides will be developed later in the project. Interviews and focus groups will be digitally recorded in audio and transcribed verbatim for analysis. The whole process will be observed and documented using field-notes during each stage, supplemented where necessary by audio-recordings and resultant transcripts. Analysis will follow the Framework Approach [17]. All data generated will be coded and analysed using the electronic database software NVivo.

### Workstream 2 (WS2)

This workstream consists of a set of surveys in which respondents are given descriptions of dental health care programmes competing for funds and are asked to rank them and then value them by stating a maximum WTP (through increased taxation per annum or voluntary contribution if the respondent does not pay tax). This approach to WTP is the most frequently used and will be referred to hereon as the standard approach. In addition to the standard approach, a new approach to eliciting WTP values which has been proposed to improve consistency, termed the ‘marginal’ or ‘incremental’ approach [18], will be trialled. The approach will be tested by randomly allocating respondents in one of the surveys to one of the two approaches, the hypothesis being that the new approach will lead to more consistent responses. The approach used for the remainder of the surveys will be determined by whichever approach proves to be more consistent and preferred is by the PBMA panel.

The surveys will be split across four waves spread over 4 months, with the same respondents being targeted at each wave. This will allow a large number of scenarios to be valued but also to allow ideas from WS1 to be fed in at various stages.

### Sample

The sample is designed to be representative of the population of England based on gender, age and socio-economic status. Where necessary, results will be weighted to account for differences in demographics of the sample versus the population. For pragmatic reasons, there will be an element of clustering of the sample based on geography.

As the data will be used for econometric modelling, sample size is dictated by the number required for sufficient power for regression models. The Events per Variable approach suggests a minimum of 10 cases per variable to be included in the regression model and with an estimated 15 variables per model and a splitting of the sample in 2 for some analysis (due to the methodological experiment), the minimum number is 300 [19]. In order to ensure a sample of 300 in the final wave, a larger sample will be required at each preceding wave, with previous experience suggesting attrition of 40% after Wave 1 and 15% after each of Waves 2 and 3 meaning an initial sample of 800 would be required. In addition previous experience suggests that certain groups are more likely to drop out so these will be oversampled in the first wave.

### Recruitment

Interviewers will approach households in 50 clusters of randomly selected Lower Super Output Areas (LSOAs), by knocking on doors from a list of all addresses in the LSOA. Interviewers will recruit one person per household and use a quota target list to ensure selection of sufficient numbers of participants with required characteristics as outlined above to ensure a final representative sample.

An incentive in the form of £10 for completing wave 1 and then £15 for completion of waves 2, 3 and 4 will be offered. Those who are interested in the study will be given a verbal and written explanation by the interviewer.

### Questionnaire design

The first survey will involve collection of demographic and dental history details using standard nationally agreed questions [20]. Participants will then be randomised to different WTP elicitation methods (standard versus incremental). Up to 8 programmes (emerging from initial interviews with the panel (Phase 1, WS1)) will then be described in detail.

For those in the “standard approach” arm, participants will be asked to rank the described programmes in order of preference and then, for each programme, WTP will be elicited initially using a shuffled bidding card method [21] and then an open-ended question asking the participant’s maximum that they are willing to pay in terms of increased taxation. For those in the “incremental” arm, the first task will also be to rank the programmes in order but WTP will then be elicited in terms of the maximum a participant is willing to pay in increased taxation to secure the 8th most preferred programme (i.e. least preferred), followed by respondents being asked to state how much extra in increased taxation they would be WTP for the 7th programme compared to the 8th. Next the extra amount for 6th programme in addition to the value for the 7th and 8th programme will be elicited, then the 5th, etc. For both approaches, where participants give a zero valuation, a separate categorical list of reasons will be given, in order to determine protest and true valuations of zero [22].

Following the first survey, the best method of standard and incremental approaches will be selected by the WS1 panel and used for subsequent surveys. Further programmes (emerging from WS1) will be analysed in three subsequent surveys conducted approximately six-monthly over 2 years, each containing up to 8 programmes.

The questionnaire has been developed specifically for this project (Additional file 2) by the research team with the input of a patient advisory group. The questionnaire was piloted with a small sample of the general public and adjusted accordingly. The scenarios to be valued will be developed as the project progresses with input from the patient advisory group as well as piloting with the general public (these are therefore not available for inclusion in Additional file 1).

### Survey delivery

The data will be collected via a series of surveys delivered over a 2 year time frame. For Wave 1, the interview will take place face to face using a computer based interface completed by the participant with support from the interviewer. For waves 2–4, the option of online completion will be offered to participants in addition to face-to-face in-home completion as per wave 1.

### Data analysis

As well as appropriate descriptive statistics (weighted where necessary for representativeness and ability to pay), the determinants of WTP will be determined using econometric analysis. The precise modelling will depend on the nature of the data but may include two-part models, tobits and Heckman Selection models. Appropriate sensitivity analysis will be performed.

## Discussion

This study will lead to a development of the PBMA method and an understanding of the use of WTP in PBMA. If the recommendations made in this study are accepted, then there should be a direct improvement in the use of resources in NHS dentistry in England. However, as with any study with a wide scope, there are certain challenges. These challenges are engagement of stakeholders with the PBMA process, reluctance to accept the recommendations by policy makers, the volume of qualitative data gathered and problems associated with the WTP elicitation.

In order to maximise potential engagement, the research team have spent time embedded within stakeholder organisations, especially NHS England and Public Health England working with those who will be approached in recruitment. This has ensured that the project is designed in such a way as to best fit with the organisations and individuals who will need to be involved but has also ensured that a level of trust and understanding has been built between the research team and the stakeholders. In a similar way, this embedding with NHS England, in particular with the key policy makers in the Office of the Chief Dental Officer, has also helped to shape the direction and design of the study to ensure the maximum likelihood of acceptance of the recommendations. Although the policy makers will be kept informed of the study progress in order to maintain interest, the embedding of the research team will be stopped at the beginning of the study and the policy makers will not have any influence on the work of the panel, to ensure an independent, rigorous process is followed.

The volume of data likely to be generated is large and this is part of the reason for choosing the Framework approach to analysis as it is well suited to managing large amounts of data but is also recommended for use where influence on policy is key. In addition, the division of data into 3 phases is an important way of managing the data, allowing analysis to be undertaken within each phase, although overall analysis bringing together findings from across the three themes will be important too.

Finally, the WTP elicitation method has been criticised for being too hypothetical and too vulnerable to bias introduced by the actual elicitation method used as well as ability to pay influencing values [21, 23, 24]. Best practice methods in terms of using a shuffled payment care approach [21], and a cheap talk script [25] or similar appropriate method of reducing hypothetical bias will be used. The use of a tax payment vehicle for a genuine set of services and the use of the values in real life policy decisions will also aid in ensuring hypothetical bias is minimised. Finally, the influence of ability to pay on WTP will be studied and if necessary, weighting will be applied.

## Additional files

Additional file 1: Topic Guide for Workstream 1, Phase 1 Interviews. Draft topic guide for pre-PBMA interviews with dental commissioners. (DOCX 21 kb)

Additional file 2: Questionnaire for Workstream 2. Questionnaire without scenarios (note that the questionnaire will be delivered electronically, using display techniques that cannot be replicated in file format, so this is detailed in the document). (DOC 98 kb)

## Abbreviations

CDPH: Consultants in Dental Public Health
LDN: Local dental network
NHS: National Health Service
PAR: Participatory action research
PBMA: Programme budgeting marginal analysis
WS1: Workstream 1
WS2: Workstream 2
WTP: Willingness to pay
