# Supplementary material for: Resource allocation in NHS dentistry: recognition of societal preferences (RAINDROP): study protocol
Source: BMC Health Serv Res. 2018 Jun 22;18:487. doi: 10.1186/s12913-018-3302-8 (PMC6013861; doi:10.1186/s12913-018-3302-8)
Supplement: Supplementary file 1 — Topic Guide for Workstream 1, Phase 1 Interviews. Draft topic guide for pre-PBMA interviews with dental commissioners. (DOCX 21 kb) [file 12913_2018_3302_MOESM1_ESM.docx]

**RAINDROP: Resource Allocation in NHS Dentistry: Recognition of Societal Preferences**

**WS1, Phase 1 Topic Guide**

N.B. Key questions are in normal type. *Prompts to be used if necessary are in italics*

Can you tell me a bit about your previous roles relevant to commissioning?

*Previous jobs and positions in NHS administration*

*Dental experience*

*Change of roles with change of NHS configurations in 2012*

Can you outline what you do in your current role as a dental commissioner?

*Day to day work*

*Key relationships*

*Key meetings to attend*

*Strategic/specific projects involved with currently*

How would you describe the relationships in commissioning currently?

*With other commissioners inside/outside of dentistry*

*With CCGs*

*With dentists (individual and through LPNs)*

*With providers – primary/secondary care*

*With Dental Public Health*

*With Local Authorities*

*With HEE*

Thinking about dental commissioning nationally, what things work well?

*Relationships*

*Structures*

*Processes*

*Support*

*Leadership*

Thinking about dental commissioning nationally, what would you say are some of the aspects that are most frustrating or most need to change?

*Relationships*

*Structures*

*Processes*

*Support*

*Leadership*

What is the relationship and split of responsibilities between the national/central team and locally?

What do you currently feel about the balance of contracting versus commissioning in your personal role and nationally?

What would need to change to allow a move from contracting to commissioning?

*Relationships*

*Structures*

*Processes*

*Support*

*Leadership*

In your commissioning decisions, what ways do you set priorities for investment?

*Do you invest/disinvest?*

*What criteria do you judge a new scheme on?*

If you were to consider specific services or aspects of dentistry provision that should be decommissioned, scaled down, commissioned or scaled up, which would be the most important to look at?

*N.B. Try to encourage at least 5 contributions*
