# Supplementary material for: Resource allocation in NHS dentistry: recognition of societal preferences (RAINDROP): study protocol
Source: BMC Health Serv Res. 2018 Jun 22;18:487. doi: 10.1186/s12913-018-3302-8 (PMC6013861; doi:10.1186/s12913-018-3302-8)
Supplement: Supplementary file 2 — Questionnaire for Workstream 2. Questionnaire without scenarios (note that the questionnaire will be delivered electronically, using display techniques that cannot be replicated in file format, so this is detailed in the document). (DOC 98 kb) [file 12913_2018_3302_MOESM2_ESM.doc]

All questions, (including prompts for interviewers/respondents e.g.
'Tick all that apply') are formatted with the 'Question' style in blue.
All responses are listed and formatted using the 'Response' style in
red. Questions followed by a blank line are an open-ended or numeric
question. Instructions (i.e. routing instructions) are formatted using the
'Instruction' style in italic. Rating questions are simply listed with
the scale listed first followed by the responses and formatted using the
'Response' style.

PROJECT RAINDROP (STAGE 1 SURVEY)

Good morning/afternoon/evening, my name is………. and I am working for an independent research company called Qa Research on behalf of Newcastle University.

We’re recruiting people to take part in an important piece of research for the University which is about the types of services that dentists provide to patients. The research will help healthcare managers to allocate resources to dental services to reflect the needs and preferences of the population.

**The research involves taking part in 4 different surveys over an 18 month period and we’re looking for people who’d be happy to be involved over this time period and to complete all 4 surveys.**

**Is this something that you or anyone else in your household might like to be involved in?**

Yes ***– CONTINUE***

No ***– THANK & CLOSE***

**Each survey will take around 30-40 minutes to complete and we’re offering people £10 for taking part in the first survey and £5 for taking part in each of the other 3 surveys, as a thank you for giving up their time.**

**To ensure we get a good spread of people, the survey asks people to provide some basic information about themselves such as their age, gender and details about their employment. It then asks about the way in which you use dental services, and how you would normally expect to pay for them. The main part of the survey involves some exercises to understand how much people would be willing to pay for some different types of dental services and treatments.**

Would you like to take part in this study?

Before we begin, I’d like to reassure you that this interview will be carried out according to the Market Research Society’s Code of Conduct and all your answers and information you provide will be treated as anonymous and confidential in accordance with the Data Protection Act 1998.

SCREENERS

Firstly, I just need to find out a little bit about you to ensure we are talking to a good cross-section of people.

S1. May I ask how old you are?

NUMERICAL BOX

IF RESPONDENT DOES NOT WISH TO GIVE AGE CODE TO AGE BANDS

16-24

25-34

35-44

45-54

55-64

65 or over

S2. Code Gender (DO NOT ASK)

SINGLECODE

Male

Female

S3. Just to make sure we are speaking to people in the right area, could you please confirm your postcode?

ENTER POSTCODE

Q4a. Which of the following best describes what you’re doing at the moment? READ OUT

SINGLECODE

1. Working either full or part-time or self-employed
2. Fully retired from work
3. Disabled or long term illness, but previously worked
4. Disabled or long term illness and have never worked
5. Looking after the home, but previously worked
6. Looking after the home and have never worked
7. Unemployed
8. Full-time education at school, college or university

IF CODE 1 GOTO TO Q4B AND CLASSIFY ON BASIS OF CURRENT JOB

IF CODES 2, 3 OR 5 GOTO Q4b AND CLASSIFY ON BASIS OF LAST JOB

IF CODE 4, 6, 7 OR 8 CLASSIFY AS ‘Never worked...’ OR ‘Full-time Student’.

**Q4b. What *(TEXT SUB BASED ON RESPONSE TO Q4a:* is/was*)* your main job?**

***CODES OPEN***

**Q4c. What *(TEXT SUB BASED ON RESPONSE TO Q4a:* do/did*)* you mainly do in your job?**

**PROBE FOR QUALIFICATIONS AND TRAINING NEEDED TO DO THE JOB**

***CODES OPEN***

**Q4d. What *(TEXT SUB BASED ON RESPONSE TO Q4a:* does/did*)* the firm or organisation you *(TEXT SUB BASED ON RESPONSE TO Q4a:* work/worked*)* for mainly make or do at the place where you *(TEXT SUB BASED ON RESPONSE TO Q4a:* work/worked*)*?**

**PROBE FOR FULL DESCRIPTION SUCH AS ‘Manufacturing’, ‘Retail’ ETC.**

***CODES OPEN***

**Q4e. *(TEXT SUB BASED ON RESPONSE TO Q4a:* Are/Were*)* you working as an employee or *(TEXT SUB BASED ON RESPONSE TO Q4a:* are/were*)* you self-employed?**

***SINGLECODE***

Employee

Self-employed

***ASK Q4f AND Q4g IF ‘Employee’ AT Q4e, OTHERS GOTO Q4h***

**Q4f. In your job *(TEXT SUB BASED ON RESPONSE TO Q4a:* do/did*)* you have any formal responsibility for supervising the work of other employees?**

**Do not include supervisors of children, (e.g. teachers, nannies, childminders; supervisors of animals); or people who supervise security or buildings only (e.g. caretakers, security guards).**

***SINGLECODE***

Yes

No

**Q4g. How many people *(TEXT SUB BASED ON RESPONSE TO Q4a:* work/worked*)* for your employer at the place where you *(TEXT SUB BASED ON RESPONSE TO Q4a:* work/worked*)*. Are/Were there...READ OUT**

**INTERVIEWER (IF REQUIRED): This is the total number of employees in your workplace and we’re only interested in employees in the place that you mainly work(ed), not the whole organisation if it has a number of locations.**

***SINGLECODE***

1 to 24,

25 to 499

500 or more

***ASK Q4h AND Q4i IF ‘Self-employed’ AT Q4e, OTHERS GOTO NEXT SECTION***

**Q4h. *(TEXT SUB BASED ON RESPONSE TO Q4a:* Are/Were*)* you working on your own or *(TEXT SUB BASED ON RESPONSE TO Q4a:* do/did*)* you have employees?**

***SINGLECODE***

On own either with or without partners but no employees

With employees

***ASK Q4i IF ‘With employees’ AT Q4h, OTHERS GOTO NEXT SECTION***

**Q4i. How many people *(TEXT SUB BASED ON RESPONSE TO Q4a:* do/did*)* you employ at the place where you *(TEXT SUB BASED ON RESPONSE TO Q4a:* work/worked*)*?**

**INTERVIEWER (IF REQUIRED): This is the total number of employees in your workplace and we’re only interested in employees in the place that you mainly work(ed), not the whole organisation if it has a number of locations.**

***SINGLECODE***

1 to 24,

25 to 499

500 or more

***ALLOCATE TO NS-SEC AS FOLLOWS (THIS WILL BE CARRIED OUT IN THE OFFICE):***

***Managerial & professional occupations***

***Intermediate occupations***

***Small employers and own account workers***

***Lower supervisory and technical occupations***

***Semi-routine and routine occupations***

***Never worked and long-term unemployed***

***Full-time students***

**SECTION 1 – INTRODUCING THE ACTIVITIES & PROCEDURES**

**This section of the survey asks you to think about some different types of activities and procedures that could be provided by the NHS as part of the dental services made available to people.**

**Firstly, I’m going to read out short descriptions of 5 different activities and procedures and for each one I’ll ask if you’ve understood it based on the description.**

***RANDOMISE THE ORDER OF DENTAL INTERVENTIONS W1A TO W1E AND TEXT SUB AS APPROPRIATE BELOW – RECORD THE ORDER FOR EACH RESPONDENT***

**INTERVIEWER: HAND FLASH CARD *(TEXT SUB BASED ON RANDOMISATION:* W1A/W1B/W1C/W1D/W1E*)* TO RESPONDENT.**

**READ OUT LONG DESCRIPTION OF *(TEXT SUB BASED ON RANDOMISATION:* W1A/W1B/W1C/W1D/W1E*)*.**

**Q5a. Based on this description, do you understand this activity or procedure?**

***SINGLECODE***

Yes

No

***REPEAT THE ABOVE SECTION FOR EACH FOR THE OTHER 4 INTERVENTIONS***

**SECTION 2 – RANKING THE ACTIVITIES & PROCEDURES**

**I’m now going to ask you to think in more detail about these activities and procedures.**

**ASK ALL**

**I want you to imagine that the NHS is deciding which of these activities and procedures to fund and provide for the general public.**

**I’d like you to put the 5 activities and procedures in order, from the one you would most like to see funded to the one you would least like to see funded.**

**Q7a. So, which is the one you’d most like to see funded?**

SINGLECODE

**Q7b. And which is the next one you’d most like to see funded?**

SINGLECODE

REDUCE THE LIST BELOW SO ONLY OPTIONS THAT HAVE NOT BEEN CHOSEN ARE SHOWN

**Q7c. And which is the next one you’d most like to see funded?**

SINGLECODE

REDUCE THE LIST BELOW SO ONLY OPTIONS THAT HAVE NOT BEEN CHOSEN ARE SHOWN

**Q7d. And which is the next one you’d most like to see funded?**

SINGLECODE

REDUCE THE LIST BELOW SO ONLY OPTIONS THAT HAVE NOT BEEN CHOSEN ARE SHOWN

**Q7e. So, I have the following as the one you’d least like to see funded *(TEXT SUB BASED ON ONLY ONE NOT CHOSEN SO FAR:* W1A/W1B/W1C/W1D/W1E*).***

RECORD THE FINAL OPTION UNDER THIS QUESTION

**Just to confirm the order you’ve chosen is as follows... READ OUT**

***SHOW ORDER BASED ON RESPONSES TO Q7a-e AND MAKE SURE EACH CHOICE IS SHOWN NEXT TO THE TEXT BELOW SO IT’S CLEAR WHICH IS THE TOP AND BOTTOM RATED.***

1 – TOP RATED

2

3

4

5 – BOTTOM RATED

**INTERVIEWER: IF RESPONDENT WISHES TO CHANGE THE ORDER GO BACK AND AMEND UNTIL HAPPY.**

**SECTION 3 – ALLOCATING THE ACTIVITIES & PROCEDURES**

***RANDOMLY ALLOCATE HALF THE SAMPLE TO GROUP 1 AND HALF TO GROUP 2.***

***GROUP 1***

***IN THIS EXERCISE RESPONDENTS WILL BE ASKED TO TALK ABOUT THEIR CHOICES AT Q7A-E BASED ON THE FOLLOWING SEQUENCE;***

***5 ONLY – ASKED ABOUT FIRST***

***4 VS. 5***

***3 VS. 4***

***2 VS. 3***

***1 VS. 2 – ASKED ABOUT LAST.***

***USE THE TEXT BELOW WHEN ASKING ABOUT CHOICE 5 ONLY***

**Now, I want you to think about your bottom choice which was *(TEXT SUB Q7e:***

**INTERVIEWER: POINT TO THE RELEVANT FLASHCARDS**

**I want you to think about what you would be willing to pay to have** ***(TEXT SUB Q7e)* as a service provided by the NHS**.

**As the NHS is paid for out of general taxation, we would like you to think about how much extra in tax per year your household would be willing to pay to see this service provided by the NHS.**

**SHOW ON FIRST ITERATION ONLY. INTERVIEWER (IF RESPONDENT SAYS THEY DON’T PAY TAX): I’d still like you to answer the question, but instead I’d like you to think about how much your household would be willing to pay as an annual voluntary contribution.**

**Just to remind you, this is purely research and you won’t personally be asked to pay anything by answering these questions.**

**Similar surveys have shown that people generally say they are willing to pay larger amounts when the payment is not real. They tend to forget that their budget is limited and that the money spent will not be available for other purchases. Therefore, please try to imagine that the payment is real.**

**For each of the amounts shown to you, ask yourself whether you would really be willing to pay it.**

**Also when you are thinking about this, we do not want you to think about how much you guess it would cost or what you have paid in the past for similar things, but just what value you put on the service yourself.**

**I am going to show you a series of values. I want you to consider each amount individually and decide whether you would be willing to pay that amount as extra in tax per year for your household to have** ***(TEXT SUB Q7e)* as a service provided by the NHS**.

***CREATE THREE BOXES ON SCREEN SHOWING ‘YES – WILLING TO PAY’, ‘NO – NOT WILLING TO PAY’ AND ‘NOT SURE’.***

***THE FOLLOWING VALUES NEED TO BE RANDOMLY ORDERED AND THEN PRESENTED ELECTRONICALLY TO THE RESPONDENT IN SEQUENCE – RESPONDENT WILL THEN DRAG AND DROP EACH PRICE-POINT INTO ONE OF THE 3 BOXES – RECORD THE RANDOM ORDER FOR EACH RESPONDENT;***

***£1***

***£2.50***

***£5***

***£7.50***

***£10***

***£20***

***£30***

***£50***

***£100***

***£200***

***RECORD THE LENGTH OF TIME A RESPONDENT TAKES TO ALLOCATE EACH VALUE.***

**INTERVIEWER: HAND THE TABLET TO THE RESPONDENT – YOU MAY HELP THEM IF REQUIRED.**

**Q8a1. Please allocate each of these prices into one of the 3 boxes depending on whether you would be willing for your household to pay that amount, unwilling or you’re not sure.**

***ONCE ALL PRICE-POINTS HAVE BEEN ALLOCATED SHOW ‘*Please hand the tablet back to the interviewer*’.***

**IF ANY PRICE-POINTS ARE IN THE ‘NOT SURE’ BOX DISPLAY THEM AND SHOW 2 BOXES FOR *‘YES – WILLING TO PAY’* AND *‘NO – NOT WILLING TO PAY’*.**

**Now that you have had chance to think a little more, do you want to reconsider any of the cards you were unsure about?**

**INTERVIEWER: READ OUT THE VALUES AND RE-ALLOCATE AS REQUIRED, ENCOURAGING RESPONDENT TO ALLOCATE TO YES AND NO IF POSSIBLE.**

**Q8b1.**

**IF AT LEAST ONE VALUE PLACED IN EACH OF THE ‘YES’ AND ‘NO’ POTS AT Q8a1;**

**You have indicated that you are willing to pay between (TEXT SUB FROM Q8a1 THE HIGHEST VALUE IN BOX YES – WILLING TO PAY) and (TEXT SUB FROM Q8a THE LOWEST VALUE IN BOX NO – NOT WILLING TO PAY) in additional tax to have this activity or procedure made available.**

**IF ALL VALUES PLACED IN ‘YES’ POT AT Q8a1;**

**You have indicated you are willing to pay £200 in additional tax to have this activity or procedure made available. It may be that you would be willing to pay more than this.**

**IF ALL VALUES PLACED IN ‘NOT SURE’ POT OR ‘NO’ AND ‘NOT SURE’ AT Q8a1;**

**You’ve said that you’re unsure about paying any of the suggested amounts to have this activity or procedure made available. As the lowest amount was £1.00, it might be that you’d be prepared to pay less than £1 or you may not be willing to pay anything at all.**

**IF ALL VALUES PLACED IN ‘YES’ and ‘NOT SURE’ POTS AT Q8A1;**

**You have indicated that you are willing to pay at least (TEXT SUB FROM Q8a1 THE HIGHEST VALUE IN BOX YES – WILLING TO PAY) in additional tax to have this activity or procedure made available but you were not sure about some other amounts.**

**Are you able to give an estimate of the highest amount of extra tax per year your household would be prepared to pay, in order to have this activity or procedure made available?**

***NUMERICAL RESPONSE***

Don’t know

***IF RESPONDENT ANSWERS ‘NO – UNWILLING TO PAY’ TO ALL VALUES AT Q8a1 ASK Q8c1, OTHERS GOTO NEXT COMPARISON.***

**Q8c1. You have indicated that you are not willing to pay any amount for this activity or procedure. Which of these reasons explains why you feel like this? READ OUT**

***MULTICODE***

The activity wouldn’t benefit you or your family directly

You feel that money should be invested in other types of dental activities and procedures instead

Money should be taken from other spending on the public sector and protected for dental care

You feel that other types of people in society should pay

It should be the people who actually benefit from the activity or procedure who should pay and not everyone

Rather than charging people more tax, the NHS should be more efficient to save money

You can’t afford to pay any more tax

You’d rather pay for dental services in another way

You don’t make use of any NHS dental services, so don’t think you should pay

Other (please specify)

Don’t know

***IF A VALUE (INCLUDING ZERO) IS GIVEN AT Q8b1 USE THE TEXT BELOW TO COMPARE CHOICES 4 VS. 5***

***IF ‘DON’T KNOW’ AT Q8b1 USE THE PREVIOUS SECTION OF TEXT WHERE A COMPARISON IS NOT MADE***

**Now, I want you to think about your bottom 2 choices which were *(TEXT SUB OF RESPONSES TO Q7d AND Q7e:)* and *(TEXT SUB OF RESPONSES TO Q7d AND Q7e:)*.**

**You have indicated that you prefer *(TEXT SUB Q7d)* to *(TEXT SUB Q7e)* and that you would pay be willing to pay** ***(TEXT SUB Q8b1[[1]](#footnote-2))* to have *(TEXT SUB Q7e)* as a service provided by the NHS**.

**This time, I want you to think about what extra on top of *(TEXT SUB Q8b1)* you would be willing to pay to have** ***(TEXT SUB Q7d)* as a service provided by the NHS rather than** ***(TEXT SUB Q7e)***.

**Again, I would like you to think about how much extra tax per year your household would be willing to pay to see this service provided by the NHS.**

**INTERVIEWER (IF RESPONDENT SAYS THEY DON’T PAY TAX): I’d still like you to take part in the exercise anyway as your answers will be useful.**

**I am going to show you a series of values. I want you to consider each amount individually and decide whether you would be willing to pay that amount as extra tax per year for your household on top of the *(TEXT SUB Q8b1)* you said you would pay for *(TEXT SUB Q7e)* to have** ***(TEXT SUB Q7d)* as a service provided by the NHS rather than** ***(TEXT SUB Q7e)***.

***CREATE THREE BOXES ON SCREEN SHOWING ‘YES – WILLING TO PAY’, ‘NO – NOT WILLING TO PAY’ AND ‘NOT SURE’.***

***THE FOLLOWING VALUES NEED TO BE RANDOMLY ORDERED AND THEN PRESENTED ELECTRONICALLY TO THE RESPONDENT IN SEQUENCE – RESPONDENT WILL THEN DRAG AND DROP EACH PRICE-POINT INTO ONE OF THE 3 BOXES – RECORD THE RANDOM ORDER FOR EACH RESPONDENT;***

***£1***

***£2.50***

***£5***

***£7.50***

***£10***

***£20***

***£30***

***£50***

***£100***

***£200***

***IDEALLY, RECORD THE LENGTH OF TIME A RESPONDENT TAKES TO ALLOCATE EACH VALUE.***

**INTERVIEWER: HAND THE TABLET TO THE RESPONDENT – YOU MAY HELP THEM IF REQUIRED.**

**Q8b1. Please allocate each of these prices into one of the 3 boxes depending on whether you would be willing for your household to pay that amount, unwilling or you’re not sure.**

***ONCE ALL PRICE-POINTS HAVE BEEN ALLOCATED SHOW ‘*Please hand the tablet back to the interviewer*’.***

**IF ANY PRICE-POINTS ARE IN THE ‘NOT SURE’ BOX DISPLAY THEM AND SHOW 2 BOXES FOR *‘YES – WILLING TO PAY’* AND *‘NO – NOT WILLING TO PAY’*.**

**Now that you have had chance to think a little more, do you want to reconsider any of the cards you were unsure about?**

**INTERVIEWER: READ OUT THE VALUES AND RE-ALLOCATE AS REQUIRED, ENCOURAGING RESPONDENTS TO ALLOCATE TO YES AND NO IF POSSIBLE.**

**Q8b1.**

**IF AT LEAST ONE VALUE PLACED IN EACH OF THE ‘YES’ AND ‘NO’ POTS AT Q8a1;**

**You have indicated that you are willing to pay between (TEXT SUB FROM Q8a THE HIGHEST VALUE IN BOX YES – WILLING TO PAY) and (TEXT SUB FROM Q8a THE LOWEST VALUE IN BOX NO – NOT WILLING TO PAY) in additional tax to have this activity or procedure made available.**

**IF ALL VALUES PLACED IN ‘YES’ POT AT Q8a1;**

**You have indicated you are willing to pay £200 in additional tax to have this activity or procedure made available. It may be that you would be willing to pay more than this.**

**IF ALL VALUES PLACED IN ‘NOT SURE’ POT OR ‘NO’ AND ‘NOT SURE’ AT Q8a1;**

**You’ve said that you’re unsure about paying any of the suggested amounts to have this activity or procedure made available. As the lowest amount was £1.00, it might be that you’d be prepared to pay less than £1 or you may not be willing to pay anything at all.**

**IF ALL VALUES PLACED IN ‘YES’ and ‘NOT SURE’ POTS AT Q8A1;**

**You have indicated that you are willing to pay at least (TEXT SUB FROM Q8a1 THE HIGHEST VALUE IN BOX YES – WILLING TO PAY) in additional tax to have this activity or procedure made available but you were not sure about some other amounts.**

**Are you able to give an estimate of the highest amount of extra tax per year your household would be prepared to pay, in order to have this activity or procedure made available?**

***NUMERICAL RESPONSE***

Don’t know

***IF RESPONDENT ANSWERS ‘NO – UNWILLING TO PAY’ TO ALL VALUES AT Q8a1 ASK Q8c1, OTHERS GOTO NEXT COMPARISON***

**Q8c1. You have indicated that you are not willing to pay any amount for this activity or procedure. Which of these reasons explains why you feel like this? READ OUT**

***MULTICODE***

The activity wouldn’t benefit you or your family directly

You feel that money should be invested in other types of dental activities and procedures instead

Money should be taken from other spending on the public sector and protected for dental care

You feel that other types of people in society should pay

It should be the people who actually benefit from the activity or procedure who should pay and not everyone

Rather than charging people more tax, the NHS should be more efficient to save money

You can’t afford to pay any more tax

You’d rather pay for dental services in another way

You don’t make use of any NHS dental services, so don’t think you should pay

Other (please specify)

Don’t know

***REPEAT THE TEXT ABOVE WHEN COMPARING CHOICES 3 VS. 4 – CHANGE TEXT IN FIRST LINE TO ‘...your next two choices...’.***

***REPEAT THE TEXT ABOVE WHEN COMPARING CHOICES 2 VS. 3 – CHANGE TEXT IN FIRST LINE TO ‘...your next two choices...’.***

***REPEAT THE TEXT ABOVE WHEN COMPARING CHOICES 2 VS. 1 – CHANGE TEXT IN FIRST LINE TO ‘...your top two choices...’.***

**Q8d1. Thinking about the values you said you would pay, how confident are you that you’d actually be willing to pay this amount to have the activity or procedure made available in real life? READ OUT**

***SINGLECODE***

Very confident

Fairly confident

Not very confident

Not at all confident

Don’t know

***GROUP 2***

***IN THIS EXERCISE RESPONDENTS WILL BE ASKED TO TALK ABOUT THEIR CHOICES AT Q7A-E BASED ON THE FOLLOWING SEQUENCE;***

***5 ONLY – ASKED ABOUT FIRST***

***4 ONLY***

***3 ONLY***

***2 ONLY***

***1 ONLY – ASKED ABOUT LAST.***

***USE THE TEXT BELOW WHEN ASKING ABOUT CHOICE 5 ONLY***

**Now, I want you to think about your bottom choice which was *(TEXT SUB Q7e:*.**

**I want you to think about what you would be willing to pay to have** ***(TEXT SUB Q7e)* as a service provided by the NHS**.

**As the NHS is paid for out of general taxation, we would like you to think about how much extra in tax per year your household would be willing to pay to see this service provided by the NHS.**

**SHOW ON FIRST ITERATION ONLY. INTERVIEWER (IF RESPONDENT SAYS THEY DON’T PAY TAX): I’d still like you to answer the question, but instead I’d like you to think about how much your household would be willing to pay as an annual voluntary contribution.**

**Just to remind you, this is purely research and you won’t personally be asked to pay anything by answering these questions.**

**Similar surveys have shown that people generally say they are willing to pay larger amounts when the payment is not real. They tend to forget that their budget is limited and that the money spent will not be available for other purchases. Therefore, please try to imagine that the payment is real.**

**For each of the amounts shown to you, ask yourself whether you would really be willing to pay it. Also when you are thinking about this, we do not want you to think about how much you guess it would cost or what you have paid in the past for similar things, but just what value you put on the service yourself.**

**I am going to show you a series of values. I want you to consider each amount individually and decide whether you would be willing to pay that amount as extra tax per year for your household to have** ***(TEXT SUB Q7e)* as a service provided by the NHS**.

***CREATE THREE BOXES ON SCREEN SHOWING ‘YES – WILLING TO PAY’, ‘NO – NOT WILLING TO PAY’ AND ‘NOT SURE’.***

***THE FOLLOWING VALUES NEED TO BE RANDOMLY ORDERED AND THEN PRESENTED ELECTRONICALLY TO THE RESPONDENT IN SEQUENCE – RESPONDENT WILL THEN DRAG AND DROP EACH PRICE-POINT INTO ONE OF THE 3 BOXES – RECORD THE RANDOM ORDER FOR EACH RESPONDENT;***

***£1***

***£2.50***

***£5***

***£7.50***

***£10***

***£20***

***£30***

***£50***

***£100***

***£200***

***IDEALLY, RECORD THE LENGTH OF TIME A RESPONDENT TAKES TO ALLOCATE EACH VALUE.***

**INTERVIEWER: HAND THE TABLET TO THE RESPONDENT – YOU MAY HELP THEM IF REQUIRED.**

**Q8a2. Please allocate each of these prices into one of the 3 boxes depending on whether you would be willing for your household to pay that amount, unwilling or you’re not sure.**

***ONCE ALL PRICE-POINTS HAVE BEEN ALLOCATED SHOW ‘*Please hand the tablet back to the interviewer*’.***

**IF ANY PRICE-POINTS ARE IN THE ‘NOT SURE’ BOX DISPLAY THEM AND SHOW 2 BOXES FOR *‘YES – WILLING TO PAY’* AND *‘NO – NOT WILLING TO PAY’*.**

**Now that you have had chance to think a little more, do you want to reconsider any of the cards you were unsure about?**

**INTERVIEWER: READ OUT THE VALUES AND RE-ALLOCATE AS REQUIRED, ENCOURAGING RESPONDENT TO ALLOCATE TO YES AND NO IF POSSIBLE.**

**Q8b2.**

**IF AT LEAST ONE VALUE PLACED IN EACH OF THE ‘YES’ AND ‘NO’ POTS AT Q8a1;**

**You have indicated that you are willing to pay between (TEXT SUB FROM Q8a2 THE HIGHEST VALUE IN BOX YES – WILLING TO PAY) and (TEXT SUB FROM Q8a2 THE LOWEST VALUE IN BOX NO – NOT WILLING TO PAY) in additional tax to have this activity or procedure made available.**

**IF ALL VALUES PLACED IN ‘YES’ POT AT Q8a2;**

**You have indicated you are willing to pay £200 in additional tax to have this activity or procedure made available. It may be that you would be willing to pay more than this.**

**IF ALL VALUES PLACED IN ‘NOT SURE’ POT OR ‘NO’ AND ‘NOT SURE’ AT Q8a2;**

**You’ve said that you’re unsure about paying any of the suggested amounts to have this activity or procedure made available. As the lowest amount was £1.00, it might be that you’d be prepared to pay less than £1 or you may not be willing to pay anything at all.**

**IF ALL VALUES PLACED IN ‘YES’ and ‘NOT SURE’ POTS AT Q8a2;**

**You have indicated that you are willing to pay at least (TEXT SUB FROM Q8a2 THE HIGHEST VALUE IN BOX YES – WILLING TO PAY) in additional tax to have this activity or procedure made available but you were not sure about some other amounts.**

**Are you able to give an estimate of the highest amount of extra tax per year your household would be prepared to pay, in order to have this activity or procedure made available?**

***NUMERICAL RESPONSE***

Don’t know

***IF RESPONDENT ANSWERS ‘NO – UNWILLING TO PAY’ TO ALL VALUES AT Q8a2 ASK Q8c2, OTHERS GOTO NEXT COMPARISON.***

**Q8c2. You have indicated that you are not willing to pay any amount for this activity or procedure. Which of these reasons explains why you feel like this? READ OUT**

***MULTICODE***

The activity wouldn’t benefit you or your family directly

You feel that money should be invested in other types of dental activities and procedures instead

Money should be taken from other spending on the public sector and protected for dental care

You feel that other types of people in society should pay

It should be the people who actually benefit from the activity or procedure who should pay and not everyone

Rather than charging people more tax, the NHS should be more efficient to save money

You can’t afford to pay any more tax

You’d rather pay for dental services in another way

You don’t make use of any NHS dental services, so don’t think you should pay

Other (please specify)

Don’t know

***USE THE TEXT BELOW WHEN ASKING ABOUT CHOICE 4 ONLY***

**Now, I want you to think about your next lowest choice which was *(TEXT SUB Q7d:*.**

**I want you to think about what you would be willing to pay to have** ***(TEXT SUB Q7d)* as a service provided by the NHS**.

**Again, I am going to show you a series of values. I want you to consider each amount individually and decide whether you would be willing to pay that amount as extra tax per year for your household to have** ***(TEXT SUB Q7d)* as a service provided by the NHS**.

***CREATE THREE BOXES ON SCREEN SHOWING ‘YES – WILLING TO PAY’, ‘NO – NOT WILLING TO PAY’ AND ‘NOT SURE’.***

***THE FOLLOWING VALUES NEED TO BE RANDOMLY ORDERED AND THEN PRESENTED ELECTRONICALLY TO THE RESPONDENT IN SEQUENCE – RESPONDENT WILL THEN DRAG AND DROP EACH PRICE-POINT INTO ONE OF THE 3 BOXES – RECORD THE RANDOM ORDER FOR EACH RESPONDENT;***

***£1***

***£2.50***

***£5***

***£7.50***

***£10***

***£20***

***£30***

***£50***

***£100***

***£200***

***IDEALLY, RECORD THE LENGTH OF TIME A RESPONDENT TAKES TO ALLOCATE EACH VALUE.***

**INTERVIEWER: HAND THE TABLET TO THE RESPONDENT – YOU MAY HELP THEM IF REQUIRED.**

**Q8a2. Please allocate each of these prices into one of the 3 boxes depending on whether you would be willing for your household to pay that amount, unwilling or you’re not sure.**

***ONCE ALL PRICE-POINTS HAVE BEEN ALLOCATED SHOW ‘*Please hand the tablet back to the interviewer*’.***

**IF ANY PRICE-POINTS ARE IN THE ‘NOT SURE’ BOX DISPLAY THEM AND SHOW 2 BOXES FOR *‘YES – WILLING TO PAY’* AND *‘NO – NOT WILLING TO PAY’*.**

**Now that you have had chance to think a little more, do you want to reconsider any of the cards you were unsure about?**

**INTERVIEWER: READ OUT THE VALUES AND RE-ALLOCATE AS REQUIRED, ENCOURAGING RESPONDENT TO ALLOCATE TO YES AND NO IF POSSIBLE.**

**Q8b2.**

**IF AT LEAST ONE VALUE PLACED IN EACH OF THE ‘YES’ AND ‘NO’ POTS AT Q8a2;**

**You have indicated that you are willing to pay between (TEXT SUB FROM Q8a2 THE HIGHEST VALUE IN BOX YES – WILLING TO PAY) and (TEXT SUB FROM Q8a2 THE LOWEST VALUE IN BOX NO – NOT WILLING TO PAY) in additional tax to have this activity or procedure made available.**

**IF ALL VALUES PLACED IN ‘YES’ POT AT Q8a2;**

**You have indicated you are willing to pay £200 in additional tax to have this activity or procedure made available. It may be that you would be willing to pay more than this.**

**IF ALL VALUES PLACED IN ‘NOT SURE’ POT OR ‘NO’ AND ‘NOT SURE’ AT Q8a2;**

**You’ve said that you’re unsure about paying any of the suggested amounts to have this activity or procedure made available. As the lowest amount was £1.00, it might be that you’d be prepared to pay less than £1 or you may not be willing to pay anything at all.**

**IF ALL VALUES PLACED IN ‘YES’ and ‘NOT SURE’ POTS AT Q8a2;**

**You have indicated that you are willing to pay at least (TEXT SUB FROM Q8a2 THE HIGHEST VALUE IN BOX YES – WILLING TO PAY) in additional tax to have this activity or procedure made available but you were not sure about some other amounts.**

**Are you able to give an estimate of the highest amount of extra tax per year your household would be prepared to pay, in order to have this activity or procedure made available?**

***NUMERICAL RESPONSE***

Don’t know

***IF RESPONDENT ANSWERS ‘NO – UNWILLING TO PAY’ TO ALL VALUES AT Q8a2 ASK Q8c2, OTHERS GOTO NEXT COMPARISON.***

**Q8c2. You have indicated that you are not willing to pay any amount for this activity or procedure. Which of these reasons explains why you feel like this? READ OUT**

***MULTICODE***

The activity wouldn’t benefit you or your family directly

You feel that money should be invested in other types of dental activities and procedures instead

Money should be taken from other spending on the public sector and protected for dental care

You feel that other types of people in society should pay

It should be the people who actually benefit from the activity or procedure who should pay and not everyone

Rather than charging people more tax, the NHS should be more efficient to save money

You can’t afford to pay any more tax

You’d rather pay for dental services in another way

You don’t make use of any NHS dental services, so don’t think you should pay

Other (please specify)

Don’t know

***REPEAT THE TEXT ABOVE WHEN ASSESSING CHOICES 3 – CHANGE TEXT IN FIRST LINE TO ‘...your next lowest choice...’.***

***REPEAT THE TEXT ABOVE WHEN ASSESSING CHOICES 2 – CHANGE TEXT IN FIRST LINE TO ‘...your next lowest choice...’.***

***REPEAT THE TEXT ABOVE WHEN ASSESSING CHOICES 1 – CHANGE TEXT IN FIRST LINE TO ‘...your highest choice...’.***

**Q8d2. Thinking about the values you said you would pay, how confident are you that you’d actually be willing to pay this amount to have the activity or procedure made available in real life? READ OUT**

***SINGLECODE***

Very confident

Fairly confident

Not very confident

Not at all confident

Don’t know

**SECTION 4 – USE OF DENTAL SERVICES**

**The final section asks for some details about you and your use of dentists.**

**Q10. How often do you usually visit the dentist? READ OUT**

***SINGLECODE***

At least once every six months

At least once every year

At least once every two years

Less frequently than every two years

Only when having trouble with your teeth and/or dentures

Never

Prefer not to say

Don’t know

***ASK Q11-Q13 IF EVER VISIT THE DENTIST AT Q10, OTHERS GOTO Q14***

**Q11. Which type of dental services do you usually access? READ OUT**

***SINGLECODE***

NHS

Private

Both

Don’t know

**Q12. What is the main way your dental care is paid for? READ OUT**

***MULTICODE***

Out of your pocket for NHS dental care

Out of your pocket for Private dental care

You don’t pay because you’re exempt from paying

With private insurance

Other (WRITE IN)

Prefer not to say

Don’t know

**Q13. Have you personally experienced dental pain bad enough to make you go to the dentist? READ OUT**

***SINGLECODE***

Never

Longer ago than 2 years

2 years to 6 months ago

In the last 6 months

Currently in pain

Prefer not to say

Don’t know

***ASK ALL***

**Q14. Most adults have up to 32 teeth including wisdom teeth. How many natural teeth do you have remaining?**

***SINGLECODE***

Fewer than 10

10-19

20 or more

Don’t know

**Q15. When, if ever, have you or anyone else in your household had the following dental treatments that we’ve been discussing today? READ OUT**

***SINGLECODE***

In the last 2 years

Longer ago than 2 years

Never

Don’t know

***LOOP – RANDOMISE ORDER***

**Fluoride Varnish in Nurseries**

**Root Canal Treatment**

**Extended Orthodontic Treatment (e.g. braces, teeth realignment)**

**Supervised Tooth Brushing in Schools**

**Care Home Dental Visits**

**Q16. For some time there have been discussions about how concerned the government should be about the social welfare of people. Which one of the following two opinions do you agree with most? READ OUT**

**A. The welfare state in the UK goes much too far. It takes too much care of people and deprives them of too much individual responsibility.**

**B. The welfare state in the UK is on the right track. It provides security for the elderly, the sick, and other people in distress without depriving people of individual responsibility.**

***SINGLECODE***

A

B

Don’t know

**Q17. How many children aged 0-17 are living in your household?**

**INTERVIEWER NOTE: Children must live in the household and step children are included.**

***NUMERICAL RESPONSE***

**SHOWCARD Q18**

**Q18. What is the highest level of qualification you have attained?**

***SINGLECODE***

GCSE (D-G), CSE grade 2-5,

SCE O Grades D-E/Standard Grades 4-7,

Scottish National Qualifications (Access level),

SCOTVEC National Certificate Modules

NVQ (level 1), GNVQ (Foundn),

BTEC (Intro level)

GCSE (A-C)/GCE O-level passes, CSE grade 1

SCE O Grades A-C / Standard Grades 1-3,

Scottish National Qualifications (Intermediate),

School Certificate / Matriculation

NVQ (level 2), GNVQ (Intm), BTEC (1st level)

GCE 'A'-level, AS Level, SCE Higher Grades A-C,

Scottish National Qualifications (Higher)

NVQ (level 3), GNVQ (Adv), BTEC (National level)

First degree, eg BSc, BA, MA at first degree level

NVQ (level 4), BTEC (Prof level), HND/HNC

Higher degree, eg MSc, MA, MBA, PGCE, PhD

NVQ (level 5), BTEC (Adv prof level)

None of these/Not sure

INTERVIEWER: READ OUT THE FOLLOWING QUESTION THEN HAND THE TABLET TO THE RESPONDENT TO ANSWER

**Q19a. What is your household’s income before any deductions for National Insurance, Income Tax etc.? You should include all sources of income including wages, pensions, benefits, interest on savings, and rent paid to you.**

**You may give either a weekly figure or an annual figure, which ever you prefer;**

**Weekly**

***SINGLECODE***

£0-£99

£100-£199

£200-£299

£300-£399

£400-£499

£500-£599

£600-£699

£700-£999

£1,000+

Don’t know

Prefer not to say

**Annual**

***SINGLECODE***

£0-£5,199

£5,200-£10,399

£10,400-£15,599

£15,600-£20,799

£20,800-£25,999

£26,000-£31,199

£31,200-£36,399

£36,400-£51,999

£52,000+

Don’t know

Prefer not to say

***ASK Q19b IF ‘£1,000+’ OR ‘£52,000+’ AT Q19a, OTHERS GOTO Q20***

**Q19b. Just to clarify, what is your household’s income before any deductions for National Insurance, Income Tax etc.? You should include all sources of income including wages, pensions, benefits, interest on savings, and rent paid to you.**

**Weekly**

***SINGLECODE***

£1,000 - £1,249

£1,250 - £1,731

£1,732 - £2,211

£2,212+

Don’t know

Prefer not to say

**Annual**

***SINGLECODE***

£52,000 - £64,999

£65,000 - £89,999

£90,000 - £114,999

£115,000+

Don’t know

Prefer not to say

**Q20. Finally, as I mentioned this is the first of 4 surveys which will take place over the next 18 months, so could I take your details so I can re-contact you again when the next survey is due to take place.**

Full name:

Contact number (landline):

Contact number (mobile):

Full address

Email address:

Confirm email address:

**Q21. Would you like to receive the £10 cash incentive as a thank you for taking part in this survey?**

**INTERVIEWER: Record answer below and ensure respondent signs the paper sheet if they wish to receive the incentive.**

***SINGLECODE***

Yes

No

***VALIDATION QUESTIONS***

**As part of our quality control procedures we routinely check 10% of our work, so you may receive a quick phone call to confirm that you’re happy with the interview.

Note to interviewer: If asked, validation means that a supervisor may call to check a random selection of answers. If the respondent’s information it will not be used for any other purpose nor given to any 3rd parties unless consent has been previously sought.**

Thank you very much for taking part in this survey today.

1. If no value is recorded at Q8b1 then use ‘no additional tax’. [↑](#footnote-ref-2)
